# Vaccinations during pregnancy in Germany between 2004 and 2022: a claims data analysis

**DOI:** 10.1038/s41598-026-61874-z

**Published:** 2026-08-18

**Authors:** Loviisa Mulanje, Manuela Marron, Bianca Kollhorst, Tania Schink, Wolfgang Ahrens, Lara Kim Brackmann, Ulrike Haug

**Affiliations:** 1 Department of Epidemiological Methods and Etiological Research, Leibniz Institute for Prevention Research and Epidemiology – BIPS, Bremen, Germany; 2 Faculty of Human and Health Sciences, University of Bremen, Bremen, Germany; 3 Department of Statistical Methods in Epidemiology, Leibniz Institute for Prevention Research and Epidemiology – BIPS, Bremen, Germany; 4 Department of Clinical Epidemiology, Leibniz Institute for Prevention Research and Epidemiology – BIPS, Achterstrasse 30, 28359 Bremen, Germany

**Keywords:** Maternal immunization, Health insurance data, Vaccination prevalence, Diseases, Health care, Medical research

## Abstract

Data on vaccinations during pregnancy are lacking in Germany. We aimed to assess the proportion of pregnancies with any vaccination over a 19-year period. Using health claims covering approximately 20% of the German population, we included pregnancies beginning between 2004 and 2022. We determined the proportion of pregnancies with at least one vaccination (any type and specific vaccines). Results were stratified by calendar year, maternal age, federal state of residence, and socioeconomic status. Of 2,801,552 pregnancies included overall, 423,567 (15.1%) were exposed to at least one vaccination. The proportion of pregnancies with influenza vaccination was 2% in 2004–2009 and increased after its recommendation (2010) from 7% in 2010–2014 to 19% in 2020–2022; the proportion of pregnancies with pertussis vaccination was 3% in 2015–2019 and increased after its recommendation (2020) to 37% in 2020–2022. In 2020–2022, about 0.3% of pregnancies were exposed to vaccines against mumps, rubella, and measles, which are contraindicated during pregnancy. Reasons why less than 50% of pregnancies receive recommended vaccinations should be explored, e.g., to what extent this is due to an informed decision or a lack of knowledge.

**Supplementary Information:**

The online version contains supplementary material available at 10.1038/s41598-026-61874-z.

## Introduction

The use of medicines during pregnancy always requires consideration of the benefits and potential risks to the mother and the unborn child. This also applies to vaccinations during pregnancy. From a global perspective, maternal immunization during pregnancy is important as it offers protection against vaccine-preventable infections and their complications for the mother during pregnancy, the unborn child, and the infants in the first few months of life^1–3^. Accordingly, the World Health Organization (WHO) and the Advisory Committee on Immunization Practices (ACIP) of the Centers for Disease Control and Prevention (CDC) advocate for maternal immunization against influenza, diphtheria, pertussis, tetanus, and coronavirus disease (COVID-19)^4–6^.

In countries with well-developed healthcare systems, most women have already completed age-appropriate routine vaccinations before pregnancy, but some vaccinations are still recommended during pregnancy. In Germany, the National Standing Committee on Vaccination (STIKO) has recommended vaccinations against influenza during every pregnancy since 2010, from the second trimester of pregnancy, or from the first trimester for women with comorbidities, and vaccination against pertussis since 2020 in the third trimester, or the second trimester if there is an increased probability of premature birth. Inactivated vaccines, such as those against tetanus, diphtheria, and hepatitis A and B, are acceptable during pregnancy, although not explicitly recommended by STIKO guidelines^7^. Vaccination against COVID-19 during pregnancy has been recommended by the STIKO since 2021, if the pregnant woman has no baseline immunity consisting of at least three antigenic contacts (vaccination or infection, with at least one vaccine dose)^7^. Live vaccines such as those against measles, mumps, rubella, and varicella are contraindicated during pregnancy. To date, there has been no study from Germany that has systematically investigated the proportion of pregnant women exposed to any vaccination and the different types of vaccination.

Therefore, to address this gap, the present study aims to (1) determine the proportion of pregnancies during which the mother received at least one vaccination and (2) to describe the type of vaccinations administered during pregnancy.

## Methods

### Data source

We used the so-called German Pharmacoepidemiological Research Database (GePaRD), which is based on claims data from four statutory health insurance providers in Germany and currently includes information on approximately 25 million individuals who have been insured with one of the participating providers since 2004 or later^8^. In addition to demographic data, GePaRD contains information on drug dispensations as well as outpatient (from general practitioners and specialists) and inpatient services and diagnoses. For each calendar year, there is information on approximately 20% of the general population, and all geographical regions of Germany are represented. The German health care system is characterized by statutory health insurances, covering about 90% of the general population, and a uniform access to all levels of care^9,10^.

In GePaRD, information on vaccinations reimbursed by health insurance is obtained based on codes of the German Uniform Assessment Standard (EBM), which are recorded for each administered vaccination. The COVID-19 vaccine was reimbursed by government funds rather than health insurance during our study period and could therefore not be captured in our data.

Pregnancies were identified using a validated outcome algorithm, which also provides information on the end date of the pregnancy^11^. Pregnancy onset is estimated using the expected delivery date (EDD), which is available for about 80% of pregnancies in GePaRD. The EDD is based on the last menstrual period (LMP) or early ultrasound and usually coded in the first weeks of pregnancy^12^. Pregnancy onset is estimated by subtracting 280 days from the EDD. If no (plausible) EDD is available, pregnancy onset is estimated via the median duration of pregnancies with the respective outcome.

### Study design and study population

Based on the aforementioned algorithms, we identified pregnancies and included them if they fulfilled the following criteria: (1) mother of childbearing age (13–49 years), (2) pregnancy onset between 2004 and 2022, (3) mother continuously insured from pregnancy onset to the end of pregnancy, (4) residency in Germany at pregnancy onset. A pregnancy was classified as exposed to vaccination if there was at least one EBM vaccination code at any point between the onset and the end of pregnancy.

### Data analysis

First, we determined the proportion of pregnancies with at least one vaccination overall and stratified by age group, year of pregnancy onset, federal state of residence, and two indicators of socioeconomic status (SES). One of these SES indicators uses individual information on the highest educational attainment; the other is based on the German Index of Socioeconomic Deprivation (GISD) of the district of residence, which captures socioeconomic disadvantage in defined geographic areas based on data on education, employment, and income. For each pregnancy, GISD information was linked based on the respective year and area of residence. Further information on the GISD is available at https://robert-koch-institut.github.io/German_Index_of_Socioeconomic_Deprivation_GISD/. We conducted this analysis for all pregnancies and for those ending in a live birth. In the next step, we determined the proportion of pregnancies exposed to the different types of vaccination stratified by time period of pregnancy onset: 2004–2009 (i.e. before influenza vaccination was recommended), 2010–2014 (i.e. after influenza vaccination was recommended), 2015–2019 (i.e. before pertussis vaccination was recommended), 2020–2022 (after pertussis vaccination was recommended).

We conducted further analyses focusing on pregnancies with at least one vaccination. Among these, we assessed, for each trimester and overall, the proportion of individual types of vaccinations. In the analyses for the second and third trimester, we only considered those pregnancies reaching the respective trimester in the denominator of the proportion. We conducted all statistical analyses using the software SAS version 9.4, SAS Institute Inc., Cary, North Carolina, USA.

### Code availability

The underlying code for this study is available to qualified researchers upon reasonable request to the corresponding author.

### Ethics

In Germany, the utilization of health insurance data for scientific research is regulated by the Code of Social Law. All involved health insurance providers, as well as the German Federal Office for Social Security and the Senator for Health, Women and Consumer Protection in Bremen, as their responsible authorities, approved the use of GePaRD data for this study. Informed consent for studies based on claims data is required by law unless obtaining consent appears unacceptable and would bias results, which was the case in this study. According to the Ethics Committee of the University of Bremen, studies based on GePaRD are exempt from institutional review board review.

## Results

**Fig. 1:**
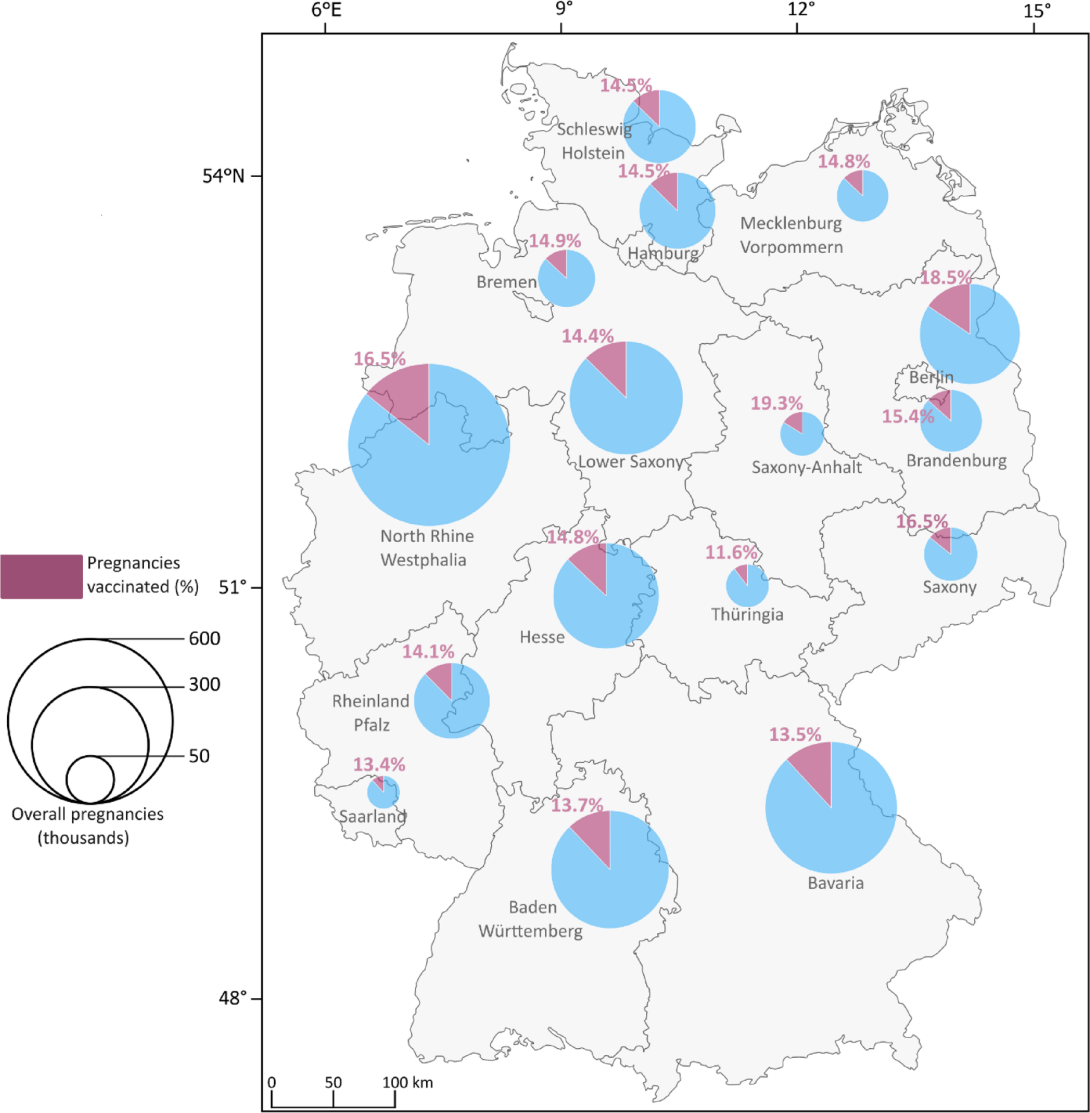
Proportion of pregnancies with at least one vaccination stratified by federal state in the period 2004 to 2022 (Please note that differences between federal states, e.g. regarding age at pregnancy onset, are not considered here. GePaRD does not cover the same proportion of pregnancies in each federal state, i.e., the numbers are not representative.) Map created using QGIS Geographic Information System version 3.42.0 (QGIS Development Team, Open Source Geospatial Foundation; https://qgis.org).

Overall, we included 2,801,552 pregnancies beginning between 2004 and 2022 (Table 1). Of these, 423,567 (15.1%) were classified as being exposed to at least one vaccination during pregnancy (Table 1). Among pregnancies ending in a live birth (N = 2,259,025), 17.4% were exposed to at least one vaccination (Supplementary Table 1). Stratification by age showed that the proportion of pregnancies exposed to at least one vaccination was highest in mothers aged 30–34 years (17.0%) and 35–39 years (16.5%), and lowest in the age group ≤ 24 years (10.2%). The proportion of pregnancies exposed to at least one vaccination increased from 3.6% for pregnancies starting between 2004 and 2009 to 43.6% for those starting between 2020 and 2022. Regarding indicators of socioeconomic status, the proportion with at least one vaccination was higher in those with a higher education (17.7%) as compared to those with basic/secondary education (11.3%) (Table 1). The proportion of pregnancies with at least one vaccination was 19.3% in Saxony-Anhalt, 18.5% in Berlin, and 16.5% in both North Rhine-Westphalia and Saxony (Fig. 1). The patterns observed for all pregnancies were similar to the patterns observed for pregnancies ending in a live birth (Supplementary Table 1 and Supplementary Fig. 1).

**Table 1:** Number and characteristics of included pregnancies overall and restricted to those with at least one vaccination in 2004–2022.

	All pregnancies	Pregnancies with ≥ 1 vaccination
N	2,801,552	423,567 (15.1)
Maternal age at pregnancy onset, n (%)		
≤ 24 years	277,961 (9.9)	28,488 (10.2)
25–29 years	691,487 (24.7)	92,877 (13.4)
30–34 years	1,034,573 (36.9)	175,735 (17.0)
35–39 years	628,312 (22.4)	103,985 (16.5)
≥ 40 years	169,219 (6.0)	22,482 (13.3)
Year of pregnancy beginning, n (%)		
2004–2009	659,101 (23.5)	23,478 (3.6)
2010–2014	739,603 (26.4)	65,226 (8.8)
2015–2019	950,057 (33.9)	137,302 (14.5)
2020–2022	452,791 (16.2)	197,561 (43.6)
Educational attainment, n (%)		
Higher education	1,669,754 (59.6)	295,571 (17.7)
Basic secondary/secondary degree	980,718 (35.0)	110,823 (11.3)
Missing	151,080 (5.4)	17,173 (11.4)
GISD*, n (%)		
Low	793,604 (28.3)	122,784 (15.5)
Medium	1,582,632 (56.5)	243,957 (15.4)
High	423,781 (15.1)	56,771 (13.4)

% in the first column refers to the total number (column %), and the % in the second column refers to the respective value for all pregnancies (row %). *German index of socioeconomic deprivation.

**Fig. 2:**
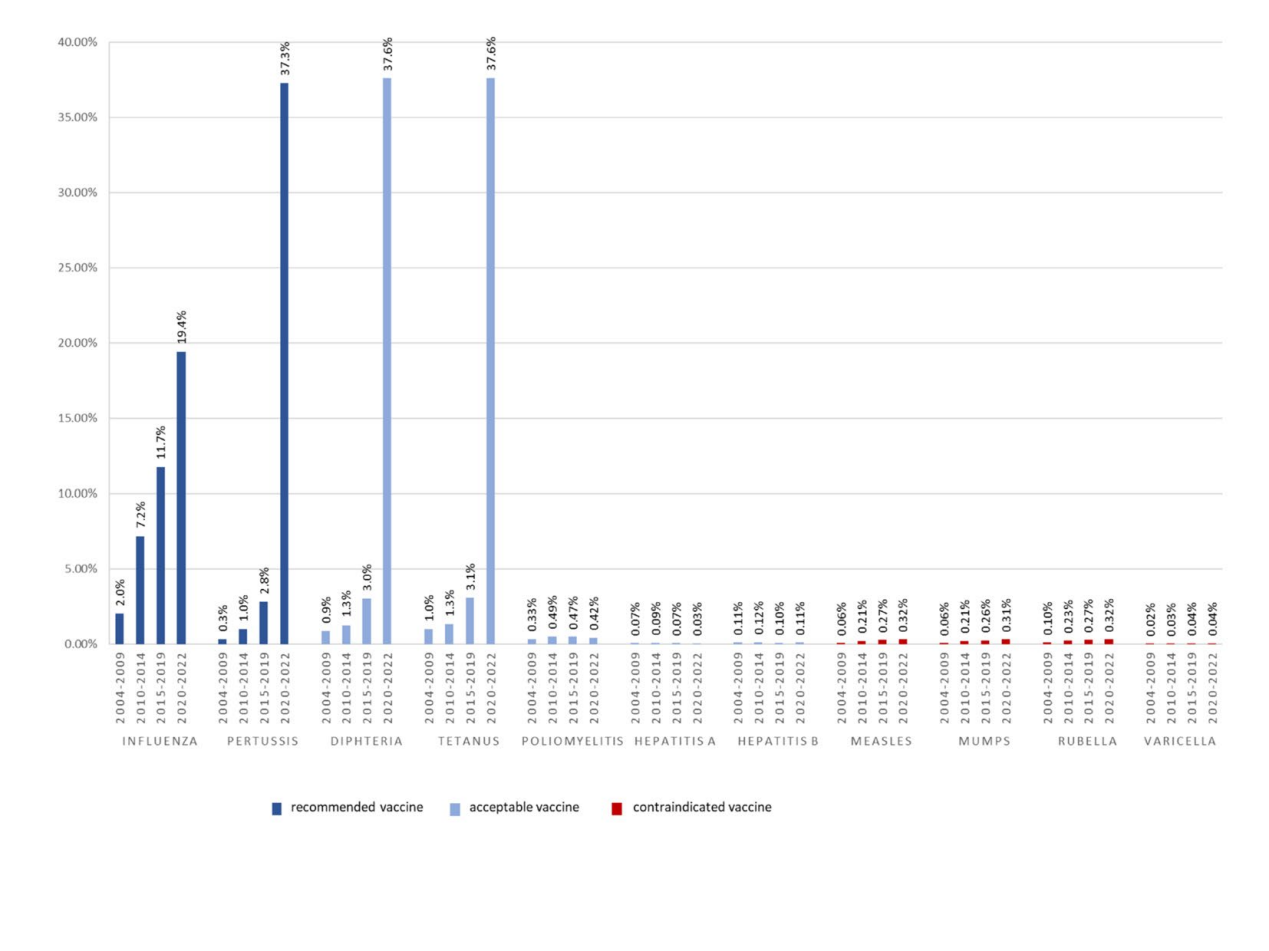
Proportion of single vaccines for all pregnancies stratified by STIKO recommendations in the period 2004 to 2022 (*Influenza recommended by STIKO since 2010, and pertussis recommended since 2020).

Figure 2 shows the proportion of pregnancies exposed to the different types of vaccinations stratified by time period. In the most recent time period (2020–2022), approximately 37% of all pregnancies were exposed to vaccines against pertussis, tetanus, and diphtheria, respectively, while in the time period 2015–2019 this applied to about 3% of pregnancies. The proportion of pregnancies with a vaccination against influenza increased from 2.0% in the period 2004–2009, 7.2% in the period 2010–2014, and 11.7% in the period 2015–2019 to 19.4% in the period 2020–2022 (Fig. 2). Overall, there were 6872 (0.2%) pregnancies exposed to a contraindicated vaccination between 2004 and 2022 (Table 2). The proportion of pregnancies with at least one contraindicated vaccination decreased during the study period (Table 2). In the most recent time period (2020–2022), about 0.3% of pregnancies were exposed to vaccines against mumps, rubella, and measles. For varicella vaccination, the proportion was 0.04%.

**Table 2:** All pregnancies with at least one vaccination: vaccines administered overall and stratified by trimester and year of pregnancy beginning.

		2004–2009	2010–2014	2015–2019	2020–2022
N (at least one vaccination)	423,567	23,478	65,226	137,302	197,561
Pregnancies with at least 1 day in the second trimester	415,744	21,762	63,276	134,786	195,920
Pregnancies with at least 1 day in the third trimester	408,265	20,935	61,581	132,007	193,742
Any vaccination, n (%)^a^					
First trimester	84,783 (20.0)	13,180 (56.1)	19,135 (29.3)	32,257 (23.5)	20,211 (10.2)
Second trimester	172,092 (41.4)	7396 (34.0)	32,568 (51.5)	69,467 (51.5)	62,661 (32.0)
Third trimester	217,808 (53.3)	3724 (17.8)	15,413 (25.0)	42,938 (32.5)	155,733 (80.4)
Any contraindicated vaccination, n (%)					
Anytime during pregnancy	6872 (1.6)	734 (3.1)	1840 (2.8)	2749 (2.0)	1549 (0.8)
First trimester	5270 (1.2)	585 (2.5)	1398 (2.1)	2104 (1.5)	1183 (0.6)
Second trimester	1286 (0.3)	118 (0.5)	352 (0.6)	548 (0.4)	268 (0.1)
Third trimester	532 (0.1)	45 (0.2)	144 (0.2)	194 (0.1)	149 (0.1)

^a^In the interpretation of shifts it is important to consider which vaccinations were recommended during the respective time periods (e.g. pertussis during the third trimester has been recommended since 2020 increasing the proportion of third-trimester vaccinations).

Among all pregnancies with at least one vaccination (any type), the proportion with at least one vaccination in the first trimester decreased over time, from 56.1% in 2004–2009 to 10.2% in 2020–2022 (Table 2). A similar pattern was observed for pregnancies ending in live births (Supplementary Table 2).

## Discussion

This study, which provides the first comprehensive overview of vaccinations during pregnancy in Germany, shows that the proportion of pregnancies with at least one vaccination has increased substantially during the past 2 decades, from below 4% in the time period 2004–2009 to about 44% in 2020–2022. This reflects that vaccinations against influenza and pertussis during each pregnancy have been recommended since 2010 and 2020, respectively. In the most recent time period, 19% of pregnancies were exposed to an influenza vaccine, and 37% to a pertussis vaccine, showing that the recommendations are often not followed, particularly for influenza. The proportion exposed to vaccines against tetanus and diphtheria was nearly equal to that of pertussis, as these vaccines are mostly given as a combination vaccine with tetanus and diphtheria. For the overall proportion of pregnancies with at least one vaccination, we did not observe major differences according to age, socioeconomic status, or region of residence.

Our findings on the uptake of influenza vaccination during pregnancy are in line with other studies from Germany. For instance, a single-center study in Bavaria utilizing computer-assisted interviews and self-reported questionnaires conducted from 2015 to 2018 reported a proportion of 13% with an influenza vaccination^13^. The proportion in our study was 12% for the period 2015–2019. Another study using German claims data reported an increase in the proportion from 9% in the 2014 influenza season to 17% in 2020^14^ i.e., the time trend is also consistent with our study. The EU Council considers pregnant women as a high-risk group for severe influenza infection and sets a target of 75% coverage of influenza vaccination during pregnancy^15^. According to the most recent European Centers for Disease Control and Prevention (ECDC) report, vaccination coverage among pregnant women was reported only from Hungary, Lithuania, Slovenia, and Spain, and ranged from 1.7 to 61.9% during the period 2020–2021, with Spain reporting the highest coverage^15^. Globally, the highest uptake of influenza vaccination in pregnancy has been reported in Spain, the United Kingdom (UK), and the United States of America (USA)^16^. In these countries, targeted pregnancy vaccination programs have been implemented, including uptake monitoring^16^, active provider engagement, reminder and recall systems, or public awareness campaigns; i.e., this goes beyond the approach of merely recommending and reimbursing influenza vaccination during pregnancy.

Regarding vaccination against pertussis, the proportion of exposed pregnancies in our study was 37% in the most recent time period (2020–2022). The increase in the uptake of vaccination against pertussis during pregnancy following its recommendation in 2020 was much sharper compared to the increase in the uptake of influenza vaccination during the past decade. The coming years will show whether there will be a further increase or whether the maximum proportion of pertussis vaccination during pregnancy has already been reached. Similar to influenza vaccination, high uptakes of pertussis vaccination during pregnancy have been reached in countries such as Spain, the UK, and the USA, with proportions of 84%, 71%, and 57% in 2019, respectively^16^.

To contextualize the observed vaccination uptake proportions in pregnancy, it is interesting to compare influenza and pertussis vaccination uptake with uptake in other population groups for whom these vaccinations are recommended in Germany. In addition to pregnant women, STIKO also recommends influenza vaccination for other high-risk and vulnerable groups, including, for example, adults aged ≥ 60 years and individuals with chronic medical conditions, with a target vaccination coverage of 75%^7^. Although influenza vaccination uptake in these groups has also remained below the target threshold, it has generally been higher than that observed among pregnant women. For example, during the 2021/22 influenza season, vaccination coverage reached 43.3% among adults aged 60 years or older and 35.4% among individuals with relevant comorbidities^17^. Pertussis vaccination coverage in the general adult population has also remained suboptimal. In 2022, 49.8% of adults aged 18 years or older had received a pertussis vaccination within the previous 10 years.

Between 2004 and 2022, we observed 6872 pregnancies exposed to at least one contraindicated vaccination, i.e., a vaccination containing a live attenuated virus. Since our data covers approximately 20% of the German population, it can be assumed that approximately 34,400 pregnancies throughout Germany were exposed to such a vaccination during this period. It cannot be ruled out that in some of these pregnancies, the pregnancy onset was not estimated correctly, and the vaccination actually took place before pregnancy. However, it is very unlikely that pregnancy onset was estimated 1 month too early, while the recommendation is that pregnancy should be avoided for at least 1 month after a vaccination with live attenuated virus due to the theoretical risk to the fetus. In practice, the actual risk following exposure to live attenuated vaccines is typically considered to be low. For instance, in a systematic review by Mangtani et al., no cases of congenital rubella syndrome were identified from several studies that evaluated the incidence of congenital rubella syndrome following inadvertent vaccination of pregnant women^18^. Similarly, in a 19-year study on varicella vaccine during pregnancy in the USA and Canada by Willis et al., no cases of congenital varicella syndrome were identified among liveborn infants of women inadvertently vaccinated against varicella^19^. However, there is a case report of a baby exposed to measles-mumps-rubella vaccination early in pregnancy, who manifested a phenotype of cardiac and neurologic defects, neurodevelopmental delay, and lymphocytopenia consistent with congenital rubella syndrome^20^.

The following aspects should be considered when interpreting our study. Strengths of our study include the large database, which is free of recall and non-responder bias. The available data allowed us to assess the uptake of all vaccinations administered during pregnancy over 19 years (except for vaccination against COVID-19). With the sophisticated algorithms developed for GePaRD, misclassification of pregnancy information, including its onset, is minimized; i.e., the proportion of pregnancies classified as exposed to the various types of information can be considered to be very valid. The agreement of the proportions observed in our study for vaccinations against influenza and pertussis with those reported based on other data sources suggests representativeness of our findings. Our study also has limitations. First, vaccination data may be incomplete before 2008, prior to the nationwide implementation of standardized EBM billing codes, which may have resulted in underestimating the proportion of pregnancies with a vaccination in the time period before 2008. Second, we did not have information on COVID-19 vaccination because it was reimbursed by governmental funds rather than by health insurance during our study period. Also, our database does not capture influenza vaccinations provided directly by employers. Since vaccinations in this setting are usually done without detailed pre-treatment consultation, we expect that most pregnant women prefer to be vaccinated by their general practitioners or gynecologists. Third, as we aimed to provide a general overview of vaccinations during pregnancy in Germany, we did not assess the seasonality of vaccination coverage. This is particularly relevant for influenza vaccination, which is recommended by STIKO during the influenza season and typically from the second trimester unless comorbidities are present. Using all pregnancies as the denominator likely underestimates coverage among those actually eligible according to these criteria. However, we do not expect that there is a relevant variation in the seasonal distribution of pregnancies across calendar years, i.e., the comparison between calendar years should not be limited. Forth, although overall representativeness of GePaRD—which covers approximately 20% of the German population—has been shown for drug prescription^8^, results in the sub-population of pregnant women might be less generalizable. This applies especially to regional differences, as GePaRD does not cover the same proportion of pregnancies in each federal state. Still, it is important to consider in this context that 90% of the German population have statutory health insurance with uniform access to all levels of care.

In conclusion, our study demonstrates that the proportion of pregnancies exposed to vaccinations has substantially increased during the past decade in Germany, following the recommendations to be vaccinated against influenza and pertussis during each pregnancy. Reasons why these proportions are still well below 50% should be explored, e.g., to what extent this is due to an informed decision or a lack of knowledge. Such studies may also assess a potential impact at the provider level and assess the role of parity on vaccination uptake during pregnancy.

## Supplementary Information

Below is the link to the electronic supplementary material.

Supplementary Material 1

## Data Availability

As we are not the owners of the data, we are not legally entitled to grant access to the data used in this study. In accordance with German data protection regulations, access to the data is granted only to employees of the Leibniz Institute for Prevention Research and Epidemiology (BIPS) on the BIPS premises and in the context of approved research projects.

## Abbreviations

ACIP: Advisory Committee on Immunization Practices
BVF: Professional Association of Gynecologists
CDC: Centers for Disease Control and Prevention
COVID-19: Coronavirus disease
EBM: German Uniform Assessment Standard
EDD: Expected date of delivery
EEA: European Economic Area
EU: European Union
ECDC: European Centers for Disease Control and Prevention
GePaRD: German Pharmacoepidemiological Research Database
GISD: German index of socioeconomic deprivation
STIKO: National Standing Committee on Vaccination
Tdap: Tetanus, diphtheria, pertussis
WHO: World Health Organization
