# Supplementary material for: Vaccinations during pregnancy in Germany between 2004 and 2022: a claims data analysis
Source: Sci Rep. 2026 Aug 18;16:25897. doi: 10.1038/s41598-026-61874-z (PMC13486490; doi:10.1038/s41598-026-61874-z)
Supplement: Supplementary file 1 [file 41598_2026_61874_MOESM1_ESM.docx]

**Supplementary Table 1: Number and characteristics of included pregnancies ending in a live birth, overall and restricted to those with at least one vaccination**

|  | **All pregnancies** | **Pregnancies with ≥ 1 vaccination** |
| --- | --- | --- |
| **N** | 2,259,025 | 393,762 (17.4) |
|  |  |  |
| **Maternal age at pregnancy onset, n (%)** |  |  |
| ≤ 24 years | 205,293 (9.1) | 25,104 (12.2) |
| 25-29 years | 580,904 (25.7) | 87,200 (15.0) |
| 30-34 years | 875,720 (38.8) | 166,488 (19.0) |
| 35-39 years | 496,818 (22.0) | 96,402 (19.4) |
| ≥ 40 years | 100,290 (4.4) | 18,568 (18.5) |
|  |  |  |
| **Year of pregnancy beginning, n (%)** |  |  |
| 2004-2009 | 555,014 (24.6) | 19,452 (3.5) |
| 2010-2014 | 598,057 (26.5) | 58,775 (9.8) |
| 2015-2019 | 760,736 (33.7) | 127,438 (16.8) |
| 2020-2022 | 345,218 (15.3) | 188,097 (54.5) |
|  |  |  |
| **Educational attainment, n (%)** |  |  |
| Higher education | 1,357,664 (60.1) | 276,110 (20.3) |
| Basic secondary/secondary degree | 785,279 (34.8) | 102,100 (13.0) |
| Missing | 116,082 (5.1) | 15,552 (13.4) |
|  |  |  |
| **GISD^*^, n (%)** |  |  |
| Low | 644,919 (28.5) | 114,227 (17.7) |
| Medium | 1,274,961 (56.4) | 227,013 (17.8) |
| High | 337,963 (15.0) | 52,478 (15.5) |

% in the first column refers to the total number (column %), and the % in the second column refers to the respective value for all pregnancies (row %). *German index of socioeconomic deprivation.

**Supplementary Table 2: Pregnancies ending in a live birth with at least one vaccination: Vaccines administered overall and stratified by trimester and year of pregnancy**

|  | **Overall (N= 2,259,025)** | **2004-2009** | **2010-2014** | **2015-2019** | **2020-2022** |
| --- | --- | --- | --- | --- | --- |
| **N** | 393,762 | 19,452 | 58,775 | 127,438 | 188,097 |
|  |  |  |  |  |  |
| **Any vaccination, n (%)** |  |  |  |  |  |
| First trimester | 68,299 (17.3) | 10,350 (53.2) | 15,219 (25.9) | 26,681 (20.9) | 16,049 (8.5) |
| Second trimester | 162,092 (41.2) | 6,366 (32.7) | 30,364 (51.7) | 65,794 (51.6) | 59,568 (31.7) |
| Third trimester | 212,310 (53.9) | 3,369 (17.3) | 14,732 (25.1) | 41,752 (32.8) | 152,457 (81.1) |
|  |  |  |  |  |  |
| **Any contraindicated vaccination, n (%)** |  |  |  |  |  |
| Anytime during pregnancy | 3,206 (0.8) | 393 (2.0) | 849 (1.4) | 1,244 (1.0) | 720 (0.4) |
| First trimester | 2,789 (0.7) | 326 (1.7) | 742 (1.3) | 1,116 (0.9) | 605 (0.3) |
| Second trimester | 171 (0.0) | 41 (0.2) | 44 (0.1) | 55 (0.0) | 31 (0.0) |
| Third trimester | 260 (0.1) | 30 (0.2) | 68 (0.1) | 76 (0.1) | 86 (0.0) |

**Supplementary Figure 1: Vaccination prevalence for pregnancies ending in live births by state in the period 2004 to 2022** (Please note that differences between federal states, e.g., regarding age at pregnancy onset, are not considered here. GePaRD does not cover the same proportion of pregnancies in each federal state, i.e., the numbers are not representative.) Map created using QGIS Geographic Information System version 3.42.0 (QGIS Development Team, Open Source Geospatial Foundation; <https://qgis.org>).
